# COVID-19 detection and disease progression visualization: Deep learning on chest X-rays for classification and coarse localization

**DOI:** 10.1007/s10489-020-01867-1

**Published:** 2020-09-12

**Authors:** Tahmina Zebin, Shahadate Rezvy

**Affiliations:** 1 School of Computing Sciences, University of East Anglia, Norwich, UK; 2 School of Science and Technology, Middlesex University London, London, UK

**Keywords:** Activation maps, COVID-19, Deep neural networks, Transfer learning

## Abstract

Chest X-rays are playing an important role in the testing and diagnosis of COVID-19 disease in the recent pandemic. However, due to the limited amount of labelled medical images, automated classification of these images for positive and negative cases remains the biggest challenge in their reliable use in diagnosis and disease progression. We implemented a transfer learning pipeline for classifying COVID-19 chest X-ray images from two publicly available chest X-ray datasets^1,2^. The classifier effectively distinguishes inflammation in lungs due to COVID-19 and Pneumonia from the ones with no infection (normal). We have used multiple pre-trained convolutional backbones as the feature extractor and achieved an overall detection accuracy of 90%, 94.3%, and 96.8% for the VGG16, ResNet50, and EfficientNetB0 backbones respectively. Additionally, we trained a generative adversarial framework (a CycleGAN) to generate and augment the minority COVID-19 class in our approach. For visual explanations and interpretation purposes, we implemented a gradient class activation mapping technique to highlight the regions of the input image that are important for predictions. Additionally, these visualizations can be used to monitor the affected lung regions during disease progression and severity stages.

## Introduction

The 2019 novel Coronavirus (COVID-19) has become a serious public health issue across the world and is approaching approximately 7.759 million cases worldwide according to the statistics of the European Centre for Disease Prevention and Control on June 14th, 2020. The COVID-19 infection may manifest itself as a flu-like illness potentially progressing to acute respiratory distress syndrome. Despite the worldwide research efforts over the past few months, early detection of COVID-19 remains a challenging issue due to limited resources and the amount of data available for research. The gold standard screening method for COVID-19 is the Reverse-Transcription Polymerase Chain Reaction (RT-PCR). Chest radiography imaging is being used as an alternative screening method and done in parallel to PCR viral testing [23]. Additionally, false negatives have been reported in PCR results due to insufficient cellular content in the sample or time-consuming nature and inadequate detection when there were positive radiological findings [3]. The accuracy of Chest X-ray (CXR) diagnosis of COVID-19 infection strongly relies on radiological expertise due to the complex morphological patterns of lung involvement which can change in extent and appearance over time. If these patterns are detected with high accuracy, it can enable rapid triaging for screening, diagnosis, and management of patients with suspected or known COVID-19 infection [16].

However, the limited number of trained thoracic radiologists limits the reliable interpretation of complex chest examinations, especially in developing countries. Deep learning techniques, in the particular Convolutional Neural Networks (CNN), have been beating humans in various tasks of computer vision and other video processing tasks in recent years. Deep learning algorithms have already been applied for the detection and classification of Pneumonia [17, 25] and other diseases on radiography. Hence, it has become the natural candidate for the analysis of CXR images to address the automated COVID-19 screening. Some recent transfer learning approaches presented in [4, 6, 8, 16, 23] applied to CXR images of patients has been showing promising results in the identification of COVID-19.

In this paper, as an effort to improve the current COVID-19 detection using a limited number of publicly available CXR dataset, we devise and implement a CXR based COVID-19 disease detection and classification pipeline using a modified VGG-16, ResNet50 [11], and a recent EfficientNetB0 [22] architecture. Following the trend from the literature, for our research, we have assembled a three-class labelled dataset with X-ray images from ‘normal’, ‘COVID-19’, ‘Pneumonia’ classes. “COVID-19 Image Data Collection” [7] is currently serving as the main source of COVID-19 CXR images at this stage. To enhance the under-represented COVID-19 class, we train a generative adversarial framework to generate synthetic COVID-19 images during our experiments. Our choice for the convolutional backbone for this research is mostly driven by their lightweight nature and their performance measures in terms of accuracy, precision, and recall performances to accurately detect COVID-19.

The remaining sections of this paper are organized as follows. In Section 2, we review the current literature on COVID-19 CXR image analysis using deep learning methods. Design insights are derived from the review of the related work and we describes the dataset for the implemented network in this section. Section 3 gives details on the proposed transfer learning architecture and discusses the necessary settings, pre-trained backbones, and procedural stages. The model performance is evaluated in Section 4 where classification results in terms of recall, precision and, overall accuracy are compared and contrasted with concurrent methods reported in the literature. The influence of model backbones on the training time, loss, and model accuracy are also discussed in this section. We also present a gradient class activation mapping (Grad-CAM) technique to monitor affected lung regions during disease progression for visual interpretation purposes. Finally, conclusions are drawn in Section 5.

## Related work and data pre-processing

Chest radiography is widely used for the detection and classification of Pneumonia and other pulmonary diseases. In the context of COVID-19 research, a closer look at the literature showed increased use of CXR images over CT scans due to potentially more data available from various sources. However, accurate annotation and analysis of radiography images require a radiology expert which requires significant expertise and processing time. To identify underlying features from radiography images for the purpose of diagnostic analysis, a series of recent studies showed promising results using state-of-the-art computational and deep learning algorithms. In Section 2.1, we review the current literature on COVID-19 CXR image analysis using deep learning methods. We will derive design insights regarding the dataset and model architecture from the review of the related work. We then describe the datasets in Section 2.2, along with the necessary pre-processing and augmentation techniques in Sections 2.3 and 2.4.

### Background: Deep learning for Chest X-ray and COVID-19 diagnosis

Convolutional neural network architecture is one of the most popular and effective approaches in the diagnosis of COVD-19 from digitized images. Several reviews have been carried out to highlight recent contributions in assembling dataset to train models for COVID-19 detection. In [6], a database of 190 COVID-19, 1345 viral Pneumonia, and 1341 normal chest X-ray images was introduced. Training and validation on four different pre-trained networks, namely, Resnet18, DenseNet201, AlexNet, and SqueezeNet for the classification of two different schemes (normal and COVID-19 Pneumonia; normal, viral Pneumonia, and COVID-19 Pneumonia). The classification accuracy for both schemes was 98.3% and 96.7% respectively. The sensitivity, specificity, and precision values were also reported.

In [12], a comparison among seven different well-known deep learning neural network architectures were presented. In the experiments, they use a small dataset with only 50 images in which 25 samples are from healthy patients and 25 from COVID-19 positive patients. In their experiments, the VGG19 and the DenseNet201 were the best performing architectures. In [23], an architecture called COVID-net is created to classify X-ray images into normal, Pneumonia, and COVID-19. Different from the previous work, they use a much larger dataset consisting of 16,756 chest radiography images across 13,645 patient cases. The authors report an accuracy of 92.4% overall and sensitivity of 80% for COVID-19.

In [8], a pre-trained ResNet50 model is fine-tuned for the problem of classifying X-ray images into normal, COVID-19, bacterial Pneumonia and viral Pneumonia. The authors report better results when compared with the COVID-net, 96.23% accuracy overall, and 100% sensitivity for COVID-19. Nevertheless, it is important to highlight that the difference in [8] that it has an extra class than [23] and the dataset consists of 68 COVID-19 radiographs from 45 COVID-19 patients, 1,203 healthy patients, 931 patients with bacterial Pneumonia and 660 patients with non-COVID-19 viral Pneumonia. Additionally, the test set has only 8 COVID-19 instances for the claim of 100% sensitivity to be generalized for a larger cohort. The author in [16] employed a light-weight implementation of a COVID-19 classifier and with an accuracy of 93.9%, COVID-19 Sensitivity of 96.8%, and positive predictive value of 100% using a flat version of EfficientNet backbone. A hierarchical version of EfficientNet was also reported with 93.5% accuracy and COVID-19 sensitivity of 80.6%. Some effort has been shown in [2] using a Decompose, Transfer, and Compose (DeTraC ) architecture to deal with any irregularities in the image dataset by investigating class boundaries. A ResNet18 based shallow learning mode was used to extract discriminating features in this implementation. The research reported an accuracy of 95.12% (with a sensitivity of 97.91%, and a specificity of 91.87%) in the detection of COVID-19 X-ray images from normal, and severe acute respiratory syndrome cases.

Having reviewed the related work, it is evident that despite the success of deep learning in the detection of Covid-19 from CXR and CT images, dealing with class imbalance, effective fine-tuning and validation of the models have not been explored. In this research, we aimed to extend the development of automated multi-class classification models based on chest X-ray images. For that, we created a balanced dataset and implemented an efficient and lightweight deep learning pipeline. We developed a Generative Adversarial Network (GAN) to generate synthetic COVID-19 data and finally, we fine-tuned and optimized the hyper-parameters to improve the performance of the model.

### Dataset description

**Fig. 1:**
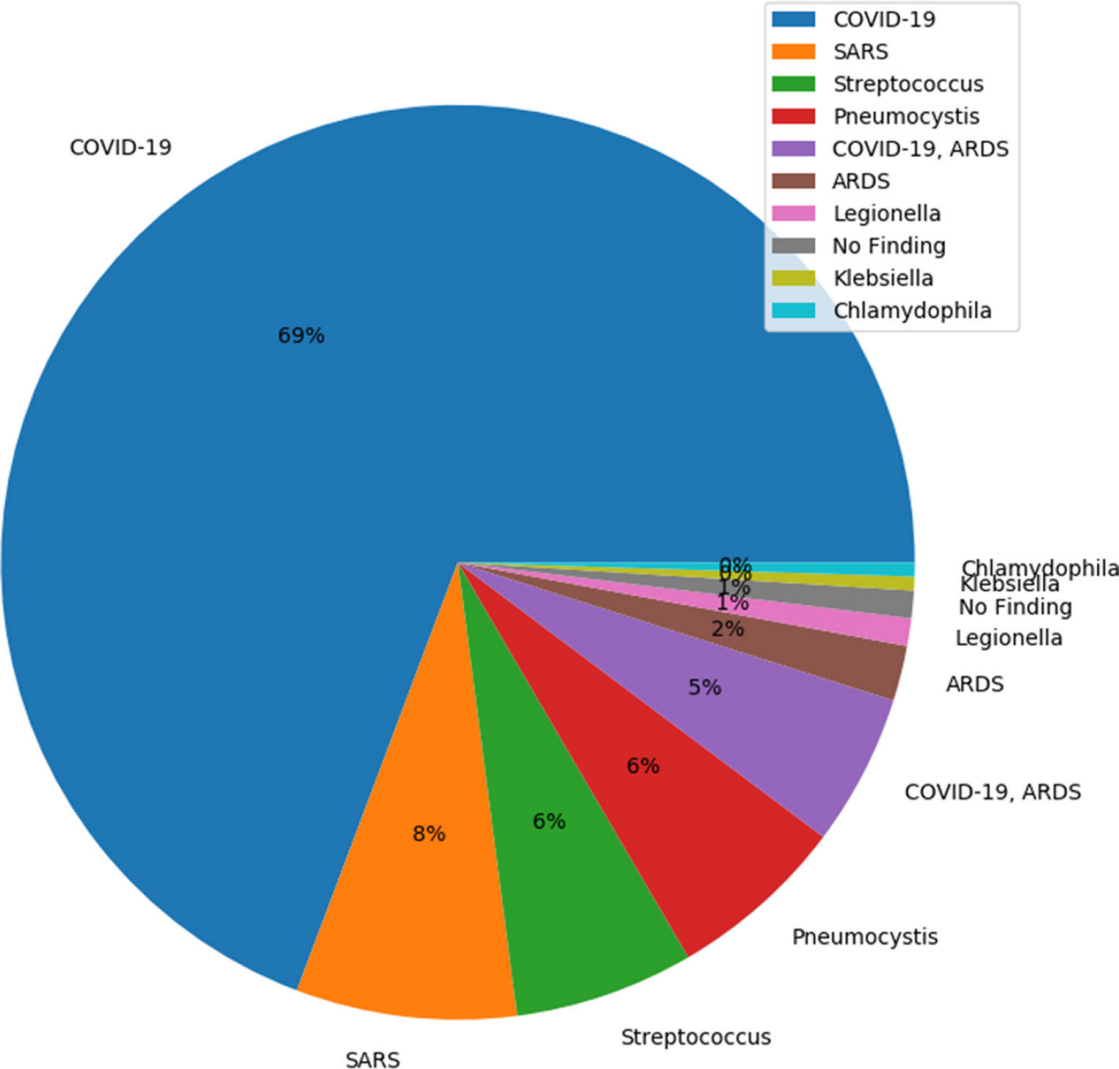
COVID-19 Image Data Collection: Image distribution as per diagnosis (69% COVID)

Following the trend of possible classes found in the literature, we have assembled a three-class dataset with labels, normal - for healthy patients; COVID-19 - for patients with COVID-19; and Pneumonia - for patients with viral and bacterial Pneumonia. Our main source of COVID-19 images was from the “COVID-19 Image Data Collection” publicly available on Github [7]. These anonymized COVID-19 images were acquired from websites of medical and scientific associations and COVID related research papers. This dataset is a constantly growing dataset and at the time of reviewing this paper in June 2020, the dataset had in total of 673 X-ray and CT images from 349 patients who were affected by COVID-19 and other diseases, such as MERS, SARS, and ARDS [3]. Figure 1 shows the percentage of image distribution as per the diagnosis, where 69% of the images had some form of COVID-19 findings. We have separated all the 202 Antero-posterior (AP) view of COVID-19 positive X-ray images from this dataset. The age group of the patients that contributed most of the positive cases were from 50 to 80 years old.

The ‘Normal’ and ‘Pneumonia’ classes for our experiments are taken from the kaggle chest-xray-Pneumonia dataset. We randomly selected 300 images for each of the classes to avoid any drastic class imbalance (dataset available at https://www.kaggle.com/paultimothymooney/chest-xray-Pneumonia). There are several other publicly available datasets of CXR images for normal, viral and bacterial Pneumonia such as the NIH Chest X-ray Dataset [13], RSNA Pneumonia Detection Challenge dataset, and a more recent COVIDx dataset from [23] which can be used for training as well.

To be noted, we selected only a small number of images for normal and Pneumonia as learning with an imbalanced dataset could produce a biased prediction model towards the classes with more samples. This made our original dataset to be consisting of 802 CXR images. 80% of the dataset is then separated as the training set, the remaining 20% of the dataset contributing as the test set. A detailed division of the dataset can be found in Table 1 and the associated code for this research can be found at https://github.com/TZebin/Covid-19-applied-Intelligence/tree/master/code.

**Table 1:** Dataset settings and other parameters

Settings	Description
Original Chest X-ray (CXR)	COVID-19: 202; Normal: 300; Pneumonia: 300
Pre-processing	Intensity normalization, class-label encoding
Training set division (80%)	COVID-19: 162; Normal: 240; Pneumonia: 240
Test set division(20%)	COVID-19: 40; Normal: 60; Pneumonia: 60
Augmentation	version1 (v1): Random rotation, width shift, height shift, horizontal flip
	version2 (v2): 100 CycleGAN synthesized image for COVID-19, followed by augmentation steps in v1
Validation set	5-fold cross-validation on the augmented training set
**Pre-trained base models:**	
VGG16	Fixed-size kernel; parameter: 138M, Input shape: 224, 224, 3
Resnet50[11]	Residual connections; 26M, Input shape: 224, 224, 3
EfficientNetB0 [22]	Mobile inverted bottleneck Convolution with depth, width, and resolution; parameter: 5.3M, Input shape: 224, 224, 3

### Pre-processing: Image resize and normalization

Each image in the assembled dataset is resized to 224x224 pixels to reduce computation time and to maintain consistency throughout our dataset. Additionally, to account for the large variability of the image appearance (brightness and contrast), depending on the acquisition source, radiation dose, etc [9, 21], an image normalization stage has been applied. This stage normalizes and scales the pixel intensities to a range of [0, 255].

### Image augmentation

To achieve robust and generalized deep learning models, large amounts of data are needed. However, medical imaging data is scarce and labelling the dataset is expensive. We applied two different versions of the augmentation technique on the dataset. In the first version, we applied image augmentation techniques [19] such as random rotation, width shift, height shift, horizontal, and vertical flip operations using the ImageDataGenerator functionality from the TensorFlow Keras framework [5, 10].

**Fig. 2:**
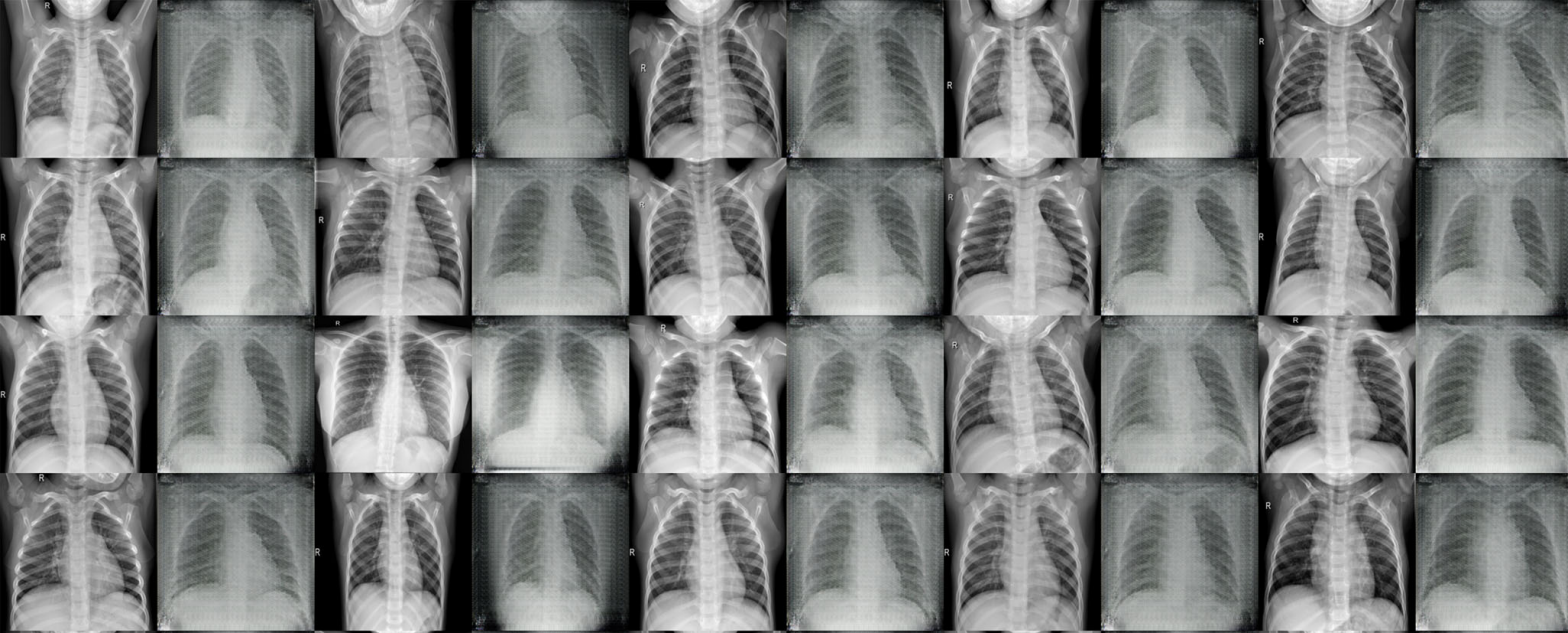
Generated images from CycleGAN for the underrepresented COVID-19 class

Nowadays, Generative adversarial networks (GAN) offer a novel method for data augmentation. Hence, we have used a CycleGAN [26] architecture for increasing the under-represented COVID-19 class images (described as version 2 for augmentation in Table 1). Utilizing the normal class from our dataset, we trained the CycleGAN to transform normal images into COVID-19 images. As a proof-of-concept at this stage, we have generated 100 synthetic COVID-19 images to add to our original training dataset. Figure 2 shows a few examples of the original and generated images side-by-side. After 5000 iterations of the generator and discriminator training, we have achieved near realistic generated CXR images, though there are shape deformations seen in some cases. To be noted, the dataset after augmentation is still quite small, hence we employed five-fold cross-validation during training to avoid the over-fitting of the model and the validation set served as a checkpoint for us to the trained model’s performance to unseen data.

## Model architecture

We implemented the COVID-19 disease detection pipeline using an adapted Convolutional Neural Network architecture and trained it in the feature representation transfer learning mode. This section gives details on the proposed transfer learning architecture and discusses the necessary settings, pre-trained backbones, and fine-tuning stages.

### Transfer learning stages

**Fig. 3:**
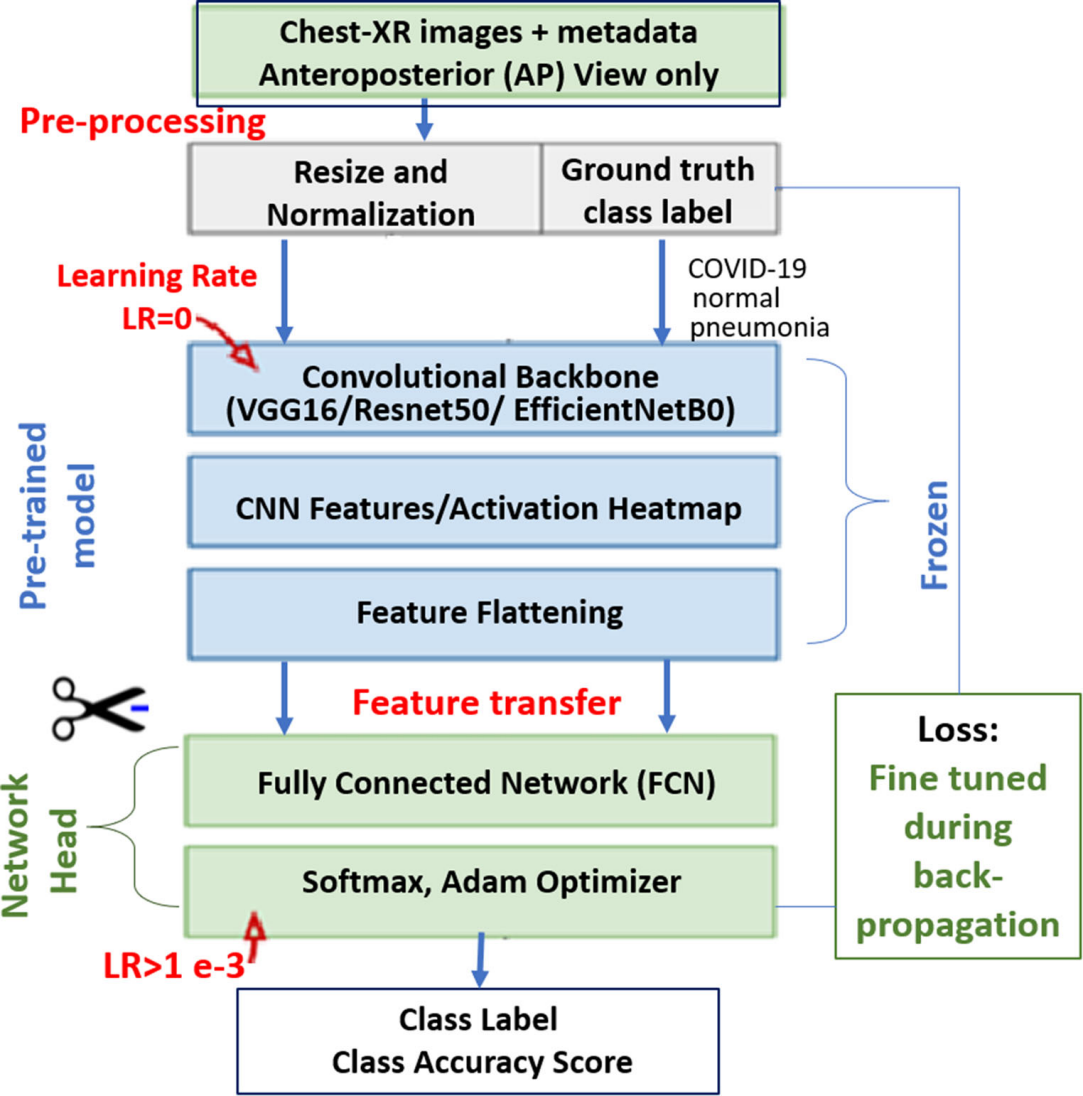
Transfer learning architecture with pre-trained convolutional backbone for COVID-19 chest X-ray classification

We effectively used a pre-trained VGG16, ResNet50, and EfficientNetB0 as our feature extractor. As all these backbones were pre-trained on huge ImageNet dataset, it has learned a good representation of low-level features like spatial, edges, rotation, lighting, shapes, and these features can be shared across to enable the knowledge transfer and act as a feature extractor for new images in different computer vision problems. As in our case, the new images have different categories from the source dataset, the pre-trained model is used to extract relevant features from these images based on the principles of transfer learning. We used TensorFlow, Keras, PyTorch, Scikit-learn, and OpenCV libraries in Python for generating various functionalities of the pipeline. Figure 3 shows an illustration of our proposed pipeline for COVID-19 chest X-ray classification.

### Pre-trained model backbone and network head removal

We removed the network head or the final layers of the pre-trained model that was initially trained on the ImageNet dataset. This stage is crucial as the pre-trained model was trained for a different classification task. The removal of network head removed weights and bias associated with the class score at the predictor layers. It is then replaced with new untrained layers with the desired number of classes in the new dataset. We adjusted a three-class network head for the COVID-19 dataset for three possible labels, namely, normal - for healthy patients, COVID-19 - for patients with COVID-19, and Pneumonia - for patients with non-COVID-19 Pneumonia.

### Fine-tuning

At the initial stage, we froze the weights of the earlier layers of the pre-trained backbone to help us extract the generic low-level descriptors or patterns from the chest X-ray image data. In the convolutional networks we used, the first few layers learn very simple and generic features that generalize to almost all types of images. As we went higher up, the features are increasingly more specific to the dataset on which the model was trained. The goal of fine-tuning is to adapt these specialized features to work with the newly fed COVID-19 dataset, rather than overwrite the generic learning.

In the feature extraction experiment, we only trained a few layers on top of a base model. The weights of the pre-trained network were not updated during training. At this stage a newly added network head or a classifier is added with the desired number of classes, and trained for adapting the weights according to the new patterns and distributions. One way to increase performance even further is to train (or “fine-tune”) the weights of the top layers of the pre-trained model alongside the training of the classifier we added. This stage forces the weights to be tuned from generic feature maps to features associated specifically with the dataset.

The training of the model has been done offline on an Ubuntu machine with Intel(R) Core i9-9900X CPU @ 3.50GHz, 62GB memory and a GeForce RTX 2060 GPU. All the models were trained for 50 epochs, fine-tuned with an Adam optimizer with a learning rate of 0.0001, Batch size of 8, and a categorical cross-entropy. To be noted, five-fold cross-validation is used during training to avoid the over-fitting of the model.

## Results and model evaluation

The main objective of our proposed architecture is to show that the pipeline we assembled, will maximize detection accuracy and minimize any false categorized COVID-19 cases. To assess the performance of the models and as a design guide for opting a backbone, we compared the training time, loss performance, and model accuracy of the VGG16, ResNet50, and EfficientNetB0 model backbones on the training set in Section 4.1. In Section 4.2, the model performance is evaluated in terms of recall, precision, and overall accuracy. These matrices are compared and contrasted with concurrent methods reported in the literature in Section 4.3. In Section 4.4, we present Grad-CAM activation maps to monitor affected lung regions during disease progression for visual explanations purposes.

### Training loss and accuracy

**Fig. 4:**
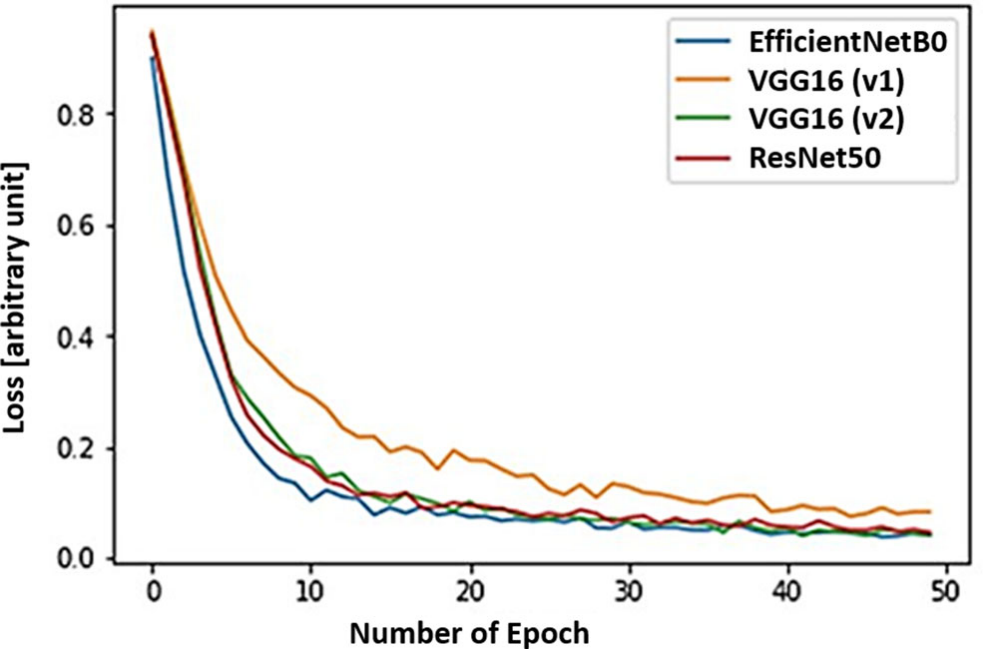
Comparative loss function on the training dataset

Figure 4 shows the change in loss function for the three convolutional models we experimented with during this research. We trained each model for 50 epochs. When the model was trained with the originally assembled three-class dataset, after traditional augmentation, the model with VGG16(v1) backbone took the longest time during training to reach an accuracy of 0.93. The VGG16(v2) is the same model trained with an enhanced version of the original dataset, where the under-represented COVID-19 class is enhanced by 100 more synthetic images generated with a CycleGAN. The training loss seemingly reached the threshold loss value within 10 epochs in this case. The realistic augmentation in the COVID-19 class definitely has increased the model’s accuracy by almost 3%. A further improvement is achieved when the backbone was replaced with ResNet50 and EfficientNetB0, with the EffiecientNetB0 being the fastest. To be noted, each epoch for the given training dataset and computational setup took about 18 seconds with 232 ms/step for a batch size of 8 and a learning rate of 0.0001. The EfficientNetB0 also achieved the best accuracy with the squeeze-and-excitation(SE) optimization stage included in its architecture.

### Model evaluation on the test dataset

If True Positive (*T*_*P*_) is the number of COVID-19 classified rightly as COVID; True Negative (*T*_*N*_) is the number of normal CXR images rightly classified normal; False Positive (*F*_*P*_) is the number of normal events misclassified as COVID-19 and non-Covid Pneumonia and False Negative (*F*_*N*_) is the number of COVID-19’s misclassified as normal or Pneumonia, we can define accuracy, recall, and the precision of a model can be defined using the following equations [20].

- Accuracy: It is an indicator of the total number of correct predictions provided by the model and defined as follows:
  1 $$ \begin{array}{@{}rcl@{}} \text{Accuracy} =\frac{T_{P}+T_{N}}{T_{P}+T_{N}+F_{P}+F_{N}}. \end{array} $$
- Recall and Precision: Two of the most commonly used performance measures, precision and recall measures are defined as follows:
  2 $$ \begin{array}{@{}rcl@{}} \text{Precision or positive predictive value} =\frac{T_{P}}{T_{P}+F_{P}}. \end{array} $$
  3 $$ \begin{array}{@{}rcl@{}} \text{Recall or Sensitivity} =\frac{T_{P}}{T_{P}+F_{N}}. \end{array} $$

**Fig. 5:**
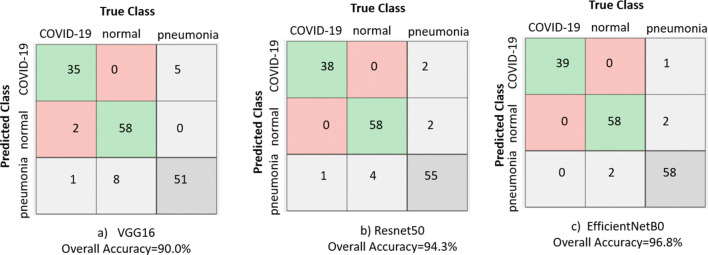
Confusion matrix and overall accuracy of three backbone models used in this research

Our results show accurate model performance with an overall detection accuracy of 90.0%, 94.3%, and 96.8% for our exemplar VGG16, ResNet50 and EfficientB0 backbones respectively on the fixed test set of 40 COVID-19, 60 Normal and 60 CXR images for the Pneumonia class. We presented the confusion matrix plot for the three backbone models under consideration in Fig. 5. The rows correspond to the predicted class (Output Class) and the columns correspond to the true class (Target Class). The diagonal cells in the confusion matrix correspond to observations that are correctly classified (*T*_*P*_ and *T*_*N*_’s). The off-diagonal cells correspond to incorrectly classified observations (*F*_*P*_ and *F*_*N*_’s). The number of observations is shown in each cell.

### Comparison with other approaches

**Fig. 6:**
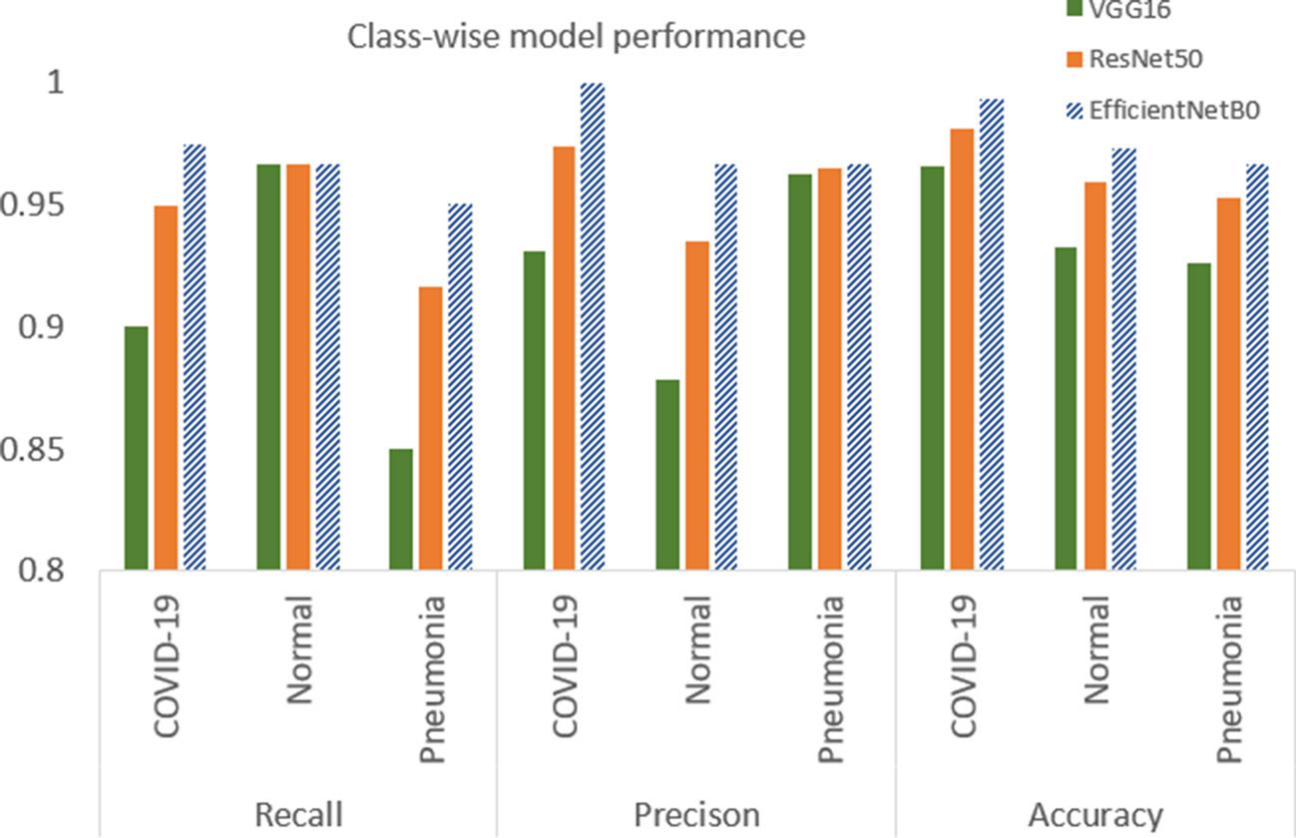
Class-wise recall, precision and accuracy comparison for the three backbone models

We have summarized a class-wise recall, precision, and accuracy performances from various experiments in Fig. 6. Table 2 showed a comparison of overall accuracy and COVID-19 precision performances with concurrent proposed approaches from the literature. As seen from our results presented in Table 2, for the VGG16 model, when the under-represented COVID-19 class is enhanced by 100 more synthetic images generated with a CycleGAN, there was a 2% improvement in overall accuracy from 0.88 to 0.90. The precision performance for the COVID-19 class has been improved from 0.88 in VGG16 (v1) to 0.93 in VGG16 (v2) through the addition of these realistically augmented COVID-19 data. When comparing this to the VGG16 model performance presented in Luz et al.[16], with a COVID-19 class data the precision value reported was 0.636. This showed a clear improvement, though the datasets used for training are not directly comparable. The VGG16 model, when saved for the inference stage, has a memory footprint of 57 megabytes with 14.7 million parameters. For the ResNet50 base model, the overall accuracy has improved to 94.3% due to a larger number of features extracted by the model, leading to a better distinction between class. This model, when serialized and saved, has a memory footprint of 97 megabytes with 23.7 million parameters. In the approach presented in [8] with ResNet50, the accuracy achieved is 96.23%, which is slightly higher than the value we achieved. However, in their test dataset, there were only 8 instances for the COVID-19 class in a four-class classification scenario, the value may not be robust and generalized for different class distribution.

**Table 2:** Class-wise precision performance comparison with other deep learning techniques in literature with our findings for COVID-19 detection

Backbone	Accuracy	COVID-19	Normal	Pneumonia
Concurrent proposed approach:				
VGG16 [16]	0.77	0.636	–	–
COVIDNet-CXR Small [23]	–	0.964	0.898	0.947
Flat - EfficientNetB0 [16]	0.90	1.0	–	–
Flat - EfficientNetB3 [16]	0.939	1.0	–	–
COVIDNet-CXR Large [23]	0.943	0.909	0.917	0.989
COVIDNet-CXR3-A[23]	–	0.979	0.921	0.903
ResNet18 [2]	0.951	0.918	0.943	–
Our results:				
VGG16 (v1 Augmentation)	0.88	0.82	0.84	0.98
VGG16 (v2 GAN Augmentation)	0.90	0.93	0.87	0.96
Resnet50 (v2 Augmentation)	0.943	0.97	0.93	0.96
EfficientNetB0 (v2 Augmented)	0.968	1.0	0.96	0.96

Our experimentation with the EfficientNetB0 base model has achieved a 96.8% overall accuracy, with a COVID-19 class precision value of 1 and a recall value of 0.975. When compared to the COVIDNet-CXR model proposed by Wang et al [23], the values were 0.909 and 0.968 respectively. Our version of EfficientNetB0 has higher precision, which is critical as the goal is to be able to detect as many positive COVID-19 cases to reduce the community spread. Using the same backbone, the EffcientNetB3 proposed by Luz et al[16] has shown a precision of 100% for the COVID-19 class, while the overall accuracy is lower than the EffcientNetB0 version we implemented. To be noted, the EffcientNetB3 model has 12.3 million parameters whereas EffcientNetB0 has 5.3 million parameters, contributing a lighter memory footprint (21 megabytes) than its scaled B3 version. Additionally, the depth, width, and resolution scaling in the EfficientNet architecture seemingly outperformed both VGG and ResNet architecture. The EfficientNetB0 also achieved the best accuracy with the squeeze-and-excitation(SE) optimization in our experiments .

### Coarse region localization map with gradient class activation

For visual explanations and interpretation purposes, we visualized the regions of the input image that are important for predictions. For this, we implemented a gradient class activation mapping (Grad-CAM) technique [18] in the pipeline to produce a coarse localization map of the highlighted regions.

**Fig. 7:**
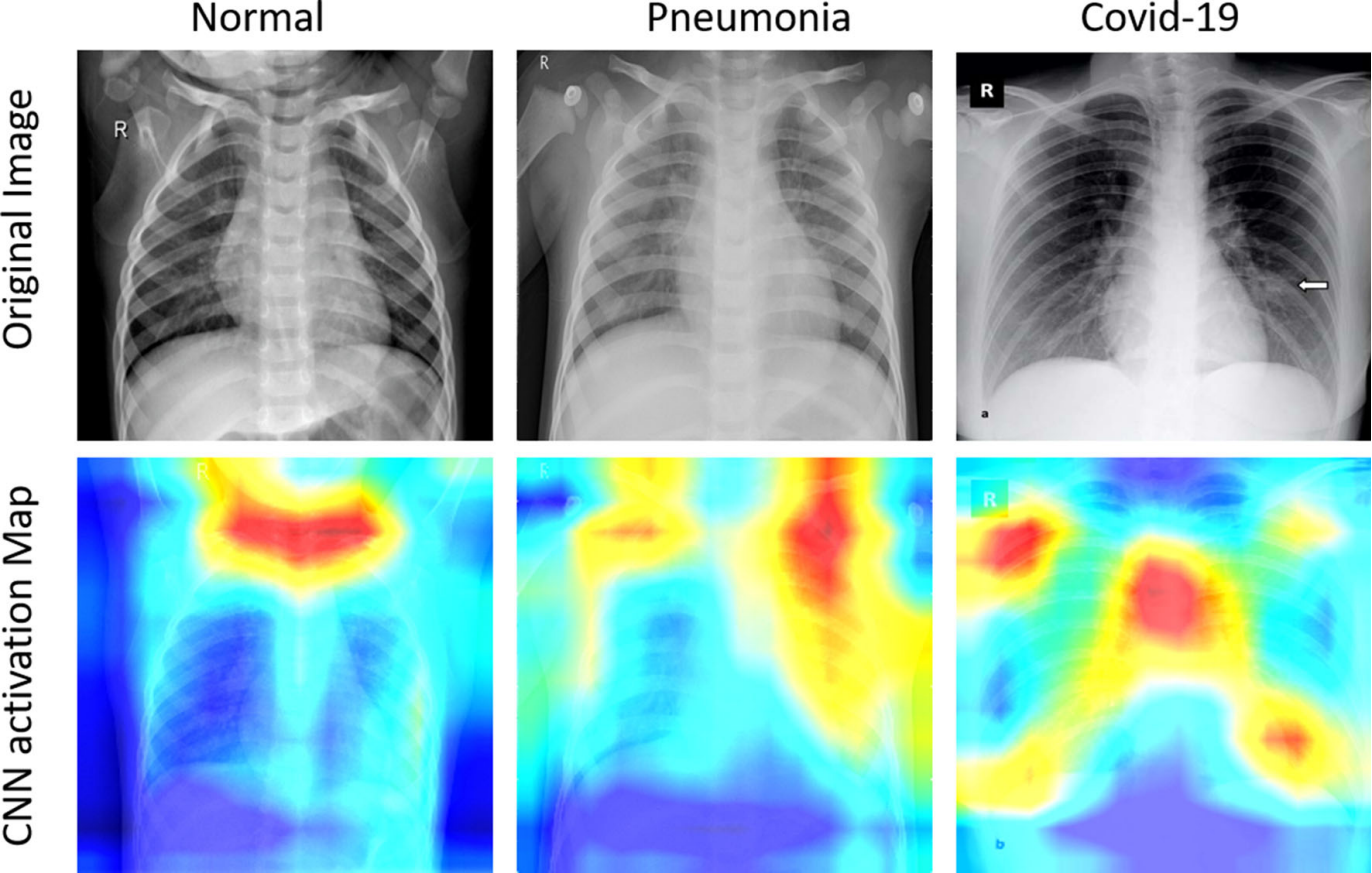
Activation map visualization for the three classes under consideration. The First column presents a healthy chest X-ray sample, the second, from a patient with Pneumonia, and the third one, from a patient with COVID-19, visualizing highly affected regions in red

**Fig. 8:**
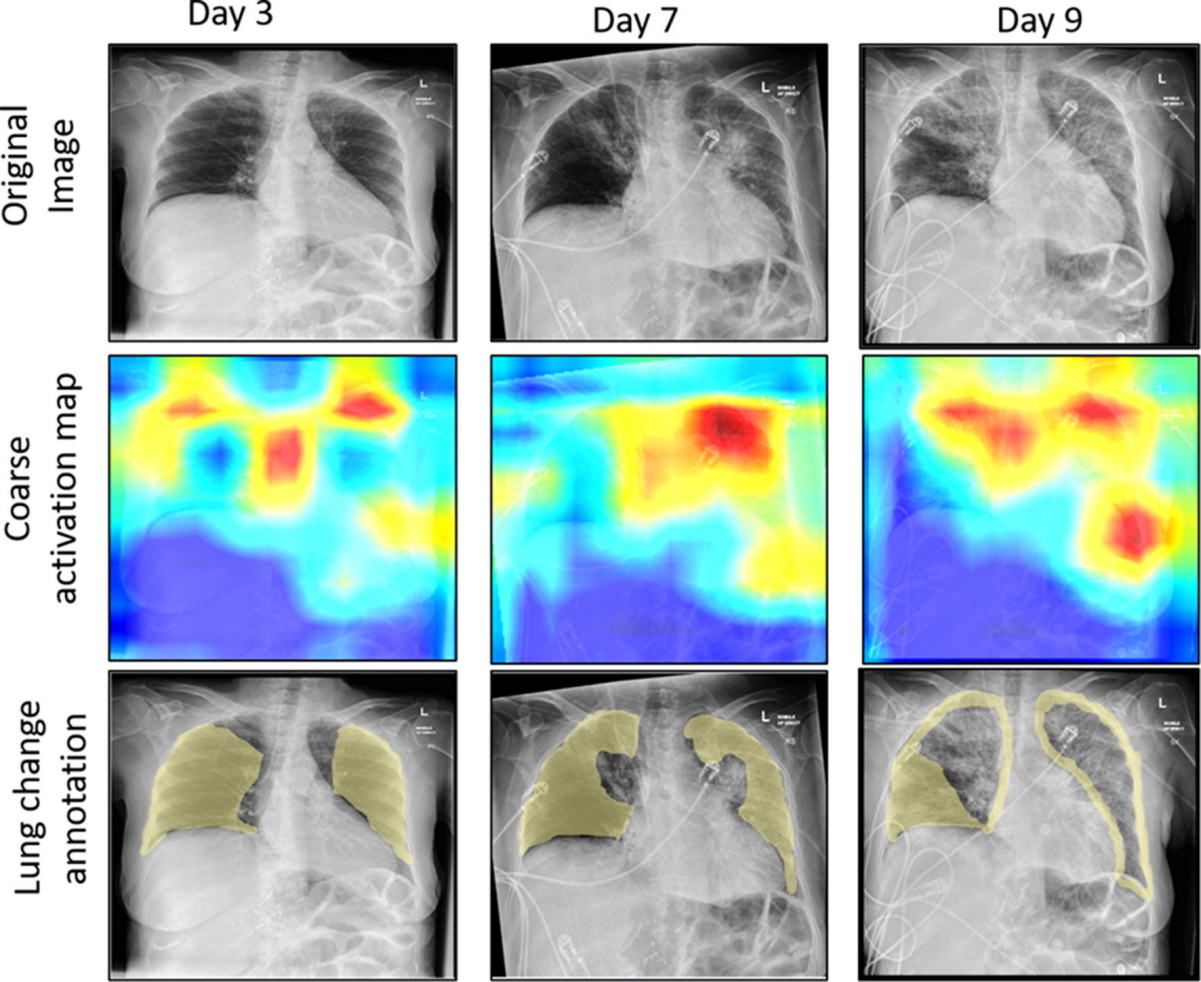
Coarse activation map visualization for a patient’s X-ray in ICU-care at day 3, 7 and 9, visualising increased inflammation (e.g. consolidations and ground-glass opacity) indicating disease severity

In Fig. 7, activation map for the three classes in our dataset is shown. The first row of Fig. 7 represents the original images, whereas the first column presents a healthy chest X-ray sample, the second shows the data from a patient with Pneumonia, and the third one, from a patient with COVID-19. The rightmost CXR taken on the patient shows bilateral patchy ground-glass opacity. These visualizations can be used to monitor affected lung regions during disease progression and severity stages. In Fig. 8, for a patient’s X-ray in ICU-care at day 3, 7 and 9, the coarse localization map showed increased inflammation indicating disease severity. There are multi-focal patch, nodular consolidations, and ground-glass opacity around the right mid to lower lung zone observed on day 9. Though clinical symptoms such as consolidations and ground-glass opacity [15] are more accurately recognizable in Computed Tomography (CT) scans, CXR images could still provide a coarse and cheap bed-side indication of such symptoms if these visualizations are enhanced by labels and clinical notes from radiologists and domain experts.

## Conclusion

Deep learning applied to chest X-rays of patients has shown promising results in the identification of COVID-19. In this research, we experimented on lightweight convolutional network architecture with three backbones (VGG-16, ResNet50, and EfficientNetB0 pre-trained on ImageNet dataset) for detecting COVID-19 and associated infection from chest X-ray images. Experiments were conducted to evaluate the convolutional neural networks performance on the traditionally augmented dataset and on an extended version of the dataset that utilized application of generative adversarial network-based augmentation using CycleGAN. Even with a limited number of images in the COVID-19 class, promising results achieved by the network on the test dataset with a recall value of over 90% and a precision value of over 93% for all the three models. We would like to emphasize on the fact that it will be possible to improve the training accuracy, sensitivity, and detection rate with more images and new data collected for the COVID-19 class. Our results also indicated the application of generative adversarial network-based augmentation techniques can contribute to accuracy improvement and can produce a more generalized and a robust model.

In future, if clinical notes and other metadata such as need for intubation and supplemental oxygen are provided, it is possible to train mixed image and metadata models. These mixed models could provide prognostic and severity predictions [7, 23] and be highly useful for risk stratification, patient management, and personalized care planning in this critical resource-constrained pandemic scenario. All models developed in this work have a memory footprint below 100 megabytes. Hence, another future direction from this research will be extending the model implementation on conventional smartphone processor to do fast and cheap on-device inference [14]. To provide a proof of concept of transferring the capability of deep learning models on mobile devices, we would like to build on our previous experience in transferring such models using the TensorFlow lite (TFlite) library [1, 24].
